# Parental Risk and Protective Factors in Child Maltreatment: A Systematic Review of the Evidence

**DOI:** 10.1177/15248380221134634

**Published:** 2022-11-30

**Authors:** Fatima Younas, Leslie Morrison Gutman

**Affiliations:** 1 University College London, London, UK

**Keywords:** child maltreatment, risk factors, protective factors, systematic review, parent

## Abstract

This study systematically reviews and synthesizes evidence on parental risk and protective factors along with identifying differences in the presence of these factors based on maltreatment type. In all, 68 quantitative, published, empirical studies were included from electronic databases for the systematic review. Quality appraisal did not exclude any studies and data were extracted from all. Results were narratively synthesized using the Risk and Resilience Ecological framework. The findings revealed more risk factors on the micro (individual and family) ecological level compared to mezzo and macro levels. At the micro level, findings mirror results of prior systematic reviews such as parental substance abuse, history of childhood maltreatment, and intimate partner violence (IPV). Social support was the most significant protective factor across all ecological levels and across all maltreatment types except child sexual abuse but differed in definition widely across studies. Physical abuse had the most risk factors unique to this type followed by neglect, and IPV was a common risk factor across all maltreatment types. Fewer studies on emotional abuse, sexual abuse, and protective factors were identified. The findings of this review delineated key parental risk and protective factors at various ecological levels along with associations between distinct factors and types of maltreatment. Interventions working with parents to reduce child maltreatment risk can use these findings to guide development of targeted programs for families based on risk and maltreatment type. For researchers, the findings can guide further investigation in under-researched areas of parental sexual and emotional abuse and protective factors.

Child maltreatment is defined as any act of commission or omission by a parent, caregiver, or another person in a custodial role which results in actual harm, potential of harm, or threat of harm to a child (CDC, 2014). Despite extensive research on its detrimental consequences (Gilbert et al., 2009), the problem of child maltreatment persists. This is partly due to heterogeneity of research findings which makes it difficult for decision-makers to reach effective conclusions to help those at risk of child maltreatment. The COVID-19 pandemic has further escalated the risk of harm to children through lockdown measures fostering social isolation, high levels of economic instability, and a rise in parents’ stress particularly for vulnerable families (Lawson et al., 2020; Pereda & Diaz-Faes, 2020). While not directly relevant, the pandemic further highlights the urgency to reduce risk of child maltreatment. To gain insight regarding buffering risk and protecting children from harm, this systematic review synthesizes evidence on four maltreatment types including physical, sexual, emotional (or psychological) abuse, and neglect which is perpetrated by one or both biological parent(s). Another type of child maltreatment, witnessing intimate partner violence (IPV), while important is not included in this review and instead is focused upon as a parental risk factor rather than a maltreatment type.

While there has been an upsurge in research on risk and protective factors for child maltreatment, immense variability in the research (including samples, methods, measures, and outcomes) makes it challenging to reach concrete conclusions and apply the best available data to real-world child maltreatment prevention methods. Some systematic reviews have focused on only one or a selection of risk factors, a specific period, or a specific population (e.g., adolescent mothers). A systematic review of protective factors for mothers at risk of inter-generational child maltreatment, for example, found that mothers’ internal capacities (e.g., self-esteem) and external resources (e.g., social support) have a buffering effect on risk of intergenerational child maltreatment (Atzl et al., 2019). However, this review focused entirely on the prenatal period and only looked at maternal history of child abuse as a risk factor and did not consider any other risk factors. Another systematic review (Timshel et al., 2017) of risk and protective factors for family violence among refugee families found that parental substance abuse, mental illness, and a parental history of childhood maltreatment played a vital role in increasing risk of child abuse and neglect. However, this study looked at all violence (e.g., domestic violence) rather than only child maltreatment, among a subset of the population (refugees), hence bringing into question generalizability to parents who are at risk of, or maltreat their children. While there have been systematic reviews of risk and protective factors for child maltreatment, no review to date and to the authors’ knowledge has exclusively focused on *parental* factors and included all types of maltreatment nor addressed specific risk and protective factors for different types of maltreatment.

With the aim of understanding risk and protective factors that can guide prevention efforts, more research on parents needs to be conducted and a focus on maltreatment type-specific factors can provide insight into the risk and protection interplay. Using the Risk and Resilience Ecological Framework (Fraser et al., 1999), this systematic review focuses on *parental* risk and protective factors. The Risk and Resilience Ecological Framework combines Bronfenbrenner’s (1979) Ecological Model and the Risk and Resilience model (Fraser, 1997), examining both risk and protective factors at the micro (individual and family), mezzo (community), and macro (national) levels. This approach acknowledges the complexities of individual influences and is useful for demarcating risk and protection based on empirical evidence.

This systematic review capitalizes on current knowledge and provides in-depth insight into the interplay of parental risk and protective factors, especially how these correspond to different types of maltreatment. This can contribute to identifying and supporting the most vulnerable parents and aid in the prevention and reduction of harm to children. There are three research questions. First, what parental factors increase the risk of child maltreatment? Second, what protective factors can buffer the risk of child maltreatment? Third, does evidence show that risk and protective factors differ based on type of child maltreatment?

## Method

A systematic review method was used to review and synthesize the vast literature on risk and protective factors in child maltreatment. Inclusion criteria for studies were limited to quantitative studies as these can provide hard numerical data and tend to employ a larger sample contributing to greater applicability of findings (see Supplemental Appendix A). Systematic reviews and meta-analysis were included in the search but only to acquire primary studies from the reviews. Once relevant primary studies were identified and obtained, the systematic reviews and meta-analyses were excluded. Studies dated from 1980 to 2018.

**Figure 1.**
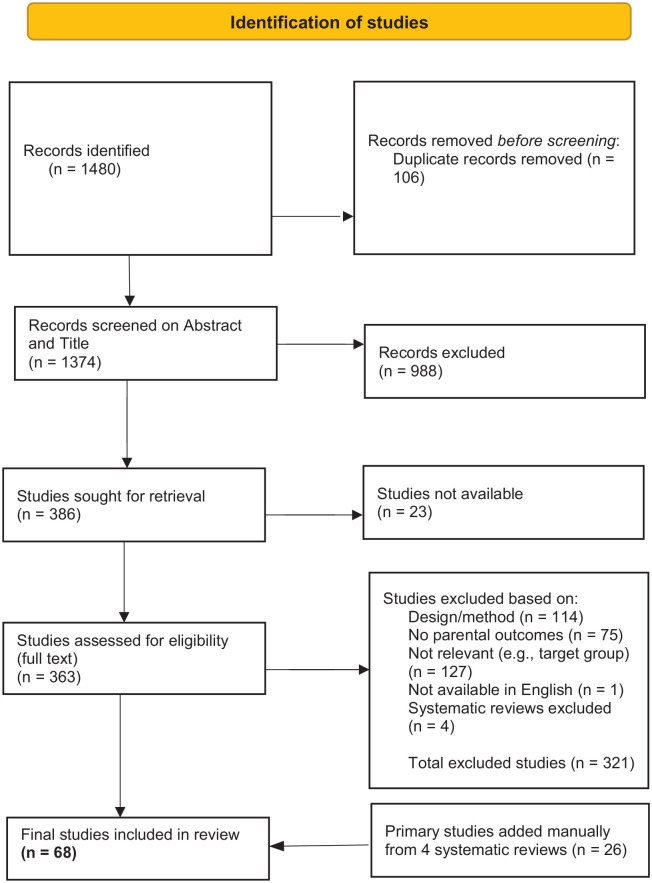
PRISMA flow chart. PRISMA = Preferred Reporting Items for Systematic Reviews and Meta Analyses.

Cochrane Library, PsychInfo, PsychExtra, Scopus, SAGE, and Web of Science were the databases searched. As a checking mechanism, the first author conducted manual searches on Child Abuse & Neglect journals (Supplemental Appendix B). Figure 1 displays a summary of the screening process using the Preferred Reporting Items for Systematic Reviews and Meta Analyses (Page et al., 2020) chart. Coding of all included studies was undertaken using EPPI-Reviewer 4 software. A data extraction form was devised to obtain relevant study characteristics (See Supplemental Appendix C).

Searches found 1,480 studies from which 106 were duplicates and 1,374 were included for title and abstract screening. From these, 1,011 studies were excluded on lack of relevance or unavailability. In all, 363 studies were eligible for full-text screening. Majority of these were excluded because the design of the study was not quantitative, the study did not include parents as perpetrators of child maltreatment, did not measure parental outcomes, or the studies were not accessible. A final 68 studies were found eligible for inclusion.

The quality assessment criteria for all included studies were adapted from The National Institute of Health and Care Excellence (NICE) guidelines (NICE, 2012) based on included study methods and summarized in one quality appraisal tool (see Supplemental Appendix D). The Grading of Recommendations, Assessment, Development and Evaluations (GRADE) approach was used to rank studies. The GRADE approach was tailored to specific criteria which only applied to methods of included studies. While this approach is primarily used in intervention evaluation studies, it is also useful for our systematic review as it addresses risk of bias and confounding which are a primary concern in the studies included in our review. Included studies were given an initial ranking of low and were then moved up to a ranking of moderate or high if there was no risk of bias, outcome measures and processes were reliable and data analysis was appropriate and valid (Kirmayr et al., 2021). Studies with a high or moderate ranking are fit for inclusion as there are no serious risks of bias that compromise a study’s quality. Any study which remains at the low ranking after consideration of quality criteria is eligible for exclusion as the study may have a high risk of bias, uninterpretable findings, and/or significant errors. A narrative synthesis approach was used to present findings on each ecological level and charts were used to illustrate significance of associations and prevalence of studies on various risk factors.

## Results

### Quality Appraisal

Following full-text screening, 68 studies were included. Quality assessment did not lead to exclusion of any studies. Table 1 shows the summary of quality appraisal results based on included studies’ ranking using the adapted GRADE approach. All 68 studies were ranked as high or moderate.

**Table 1.** Summary of Quality Appraisal Results.

No. of Studies	GRADE High	GRADE Moderate	Reason
51	✓	—	—
2		✓	Outcome measures
7		✓	Selection bias
1		✓	Non-adjustment of confounding factors
1		✓	Low survey response rate
6		✓	Issues with data analysis

### Overview of Study Characteristics

Of the 68 studies, 53 measured only risk factors, 14 measured both risk and protective factors, and one measured only protective factors. For maltreatment type, 24 studies focused on all types of child maltreatment. Five studies focused on child neglect, 19 examined child physical abuse, one emotional abuse, and one child sexual abuse. The remaining studies included more than one type of maltreatment with nine focusing on neglect and physical abuse and three on emotional and physical abuse. Six focused on three types of maltreatment; three researched child neglect, physical abuse, and sexual abuse and the other three examined child neglect, physical abuse, and emotional abuse.

There were two case–control studies, three cohort studies, 23 cross-sectional studies, two cross-study comparisons, 18 longitudinal studies, and six studies analyzing secondary data. From the 68 included studies, child maltreatment measures included all child protective service (CPS) records which was used by 16 studies, substantiated CPS reports (14), Conflict Tactic Scale (Straus et al., 1979) by six studies and the Conflict Tactic Scale Parent-Child (CTSPC; Straus et al., 1995) by seven studies. The Child Abuse Potential Inventory (Milner & Robertson, 1990) was used by 10 studies. The remaining studies used hospital records (2), national database (1), self-reports (6), referral from a child abuse program (1), and Diagnostic Interview Schedule (Robins et al., 1981) was used by one study. Five studies used more than one measure which included CTSPC and CPS reports (1), self-reports and observational measures (1), CTSPC and Multidimensional Neglectful Behavior Scale (Straus, 2006), and a mix of CPS substantiated cases, court referrals, and self-referrals was used by one study (see Supplemental Appendix E).

Data from the studies were only used as it applied to child maltreatment and parenting outcomes. There were studies which also had child outcomes and these outcomes were excluded and only parenting outcomes considered when describing study characteristics (see Supplemental Appendix E). Only the top three findings for each ecological level (micro-individual, micro-family, mezzo, and macro) are presented in detail.

### What Parental Factors Increase the Risk of Child Maltreatment?

#### Micro-level individual risk factors

**Figure 2.**
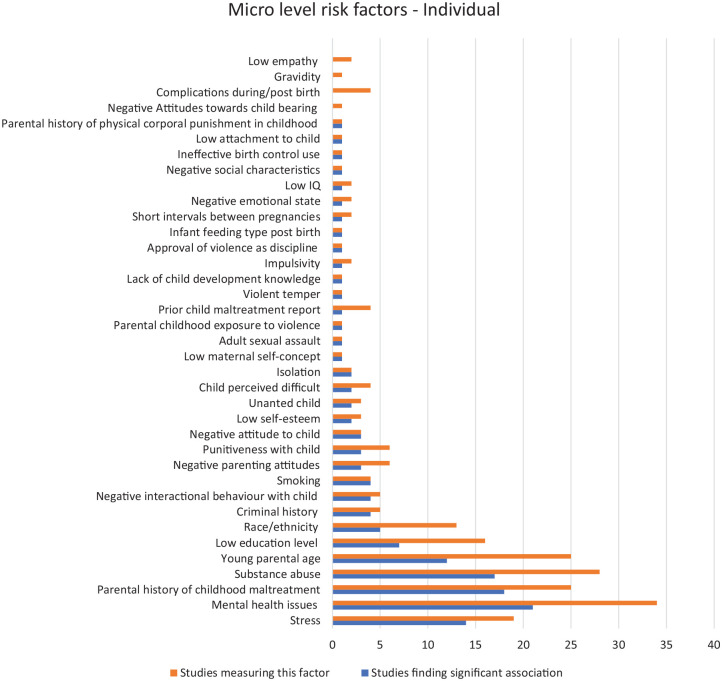
Micro-individual risk factors.

Figure 2 shows the individual-level parental risk factors studied at the micro-individual level and identifies the number of studies finding a significant association with child maltreatment. In all, 34 studies looked at parental mental health as a risk factor and 21 of these found a significant association. Anderson et al. (2018) measured psychiatric diagnosis of post-traumatic stress disorder (PTSD) and borderline personality disorder (BPD) among mothers with childhood abuse histories and found significant links to child maltreatment potential. Similarly, other studies showed depression as the most frequent mental health issue among mothers (e.g., Mash et al., 1983; Slack et al., 2011).

In all, 25 studies measured childhood history of maltreatment among parents with 21 findings showing a significant association. From these 21 studies, maternal childhood maltreatment was the focus of 14 of the studies while the remaining six included both parents’ history of abuse. Bartlett and Easterbrooks’ (2015) longitudinal study found that mothers with a history of abuse compared to those without such a history were 2.5 times more likely to neglect their infants (*p* = .038). Similarly, De Paul and Domenech’ (2000) longitudinal study on adolescent mothers found that those with memories of childhood physical abuse had a higher risk of maltreatment compared to mothers with childhood physical abuse but no *memories* of that abuse (*p* = .02). Another study (Thornberry et al., 2013) found that parents with a history of maltreatment were 2.6 times more likely to maltreat their children when the parents were aged between 21 and 30 years (odds ratio: 2.57, confidence interval: 1.47–4.50) than parents without a history.

Substance abuse was measured by 28 studies and 18 found a significant association with child maltreatment. One such study (Ricci et al., 2003) identified parental characteristics of children with abusive head trauma, with 53% having parents who abused drugs and alcohol. Another study (Fuller & Wells, 2003) found that drug and alcohol abuse was related to maltreatment recurrence (*p* = .03). Cheng and Lo (2015) also showed that recurrence of child maltreatment was positively associated with parents’ alcohol abuse (*p* < .05).

#### Micro-level family risk factors

**Figure 3.**
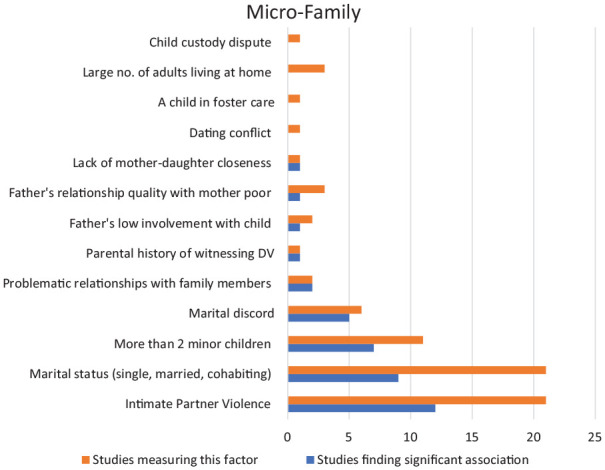
Micro-family-level risk factors.

Figure 3 displays the risk factors within the micro-family level identified among the included studies, showing the number that have a significant association with child maltreatment. Of 21 studies, nine found an association between single parenthood and child maltreatment. Dubowitz et al.’ (2011) longitudinal study explored associations of multiple level risk factors to examine antecedents and outcomes of maltreatment. In total, 224 parents were followed for 12 years by which time 43% (*n* = 97) of families had at least one child protection report and mothers of these children were less likely to be married (*p* = .07). Similarly, one longitudinal study (Fuller & Wells, 2003) investigated prognostic factors of recurrent child maltreatment among parents with substance use disorders (*n* = 95) and found that single parent families were more likely to repeatedly maltreat (*p* = .02). Marital dispute or discord was significantly associated with child maltreatment in five out of six studies. A longitudinal study (Zhao et al., 2018) with a Chinese population of neglected children (*n* = 553) found a correlation between child neglect (*p* = .03) and parental marital discord. Correspondingly, Whipple and Webster-Stratton’ (1991) study found that physically abusive mothers (*n* = 92) had higher marital distress and less satisfaction with their marital relationship (*p* = .04).

In all, 11 studies examined the number of minor children at home as a risk factor and seven found a significant association. Wu et al.’ (2004) cohort study of verified maltreatment cases found that having more than two children at home was significantly associated with infant maltreatment. One study (Wolfner & Gelles, 1993) surveyed 3,232 households and found a correlation between number of children (more than 4) and physical abuse (*p* < .01). Schick et al.’s (2015) study also found a correlation between physical abuse risk for adolescent girls which was higher among those with three or more siblings at home (*p* < .05).

#### Mezzo-level risk factors

Economic deprivation was the most prevalent risk factor associated with child maltreatment at the mezzo level. Low household income, low financial resources, poverty, and welfare receipt encompassed economic deprivation and were linked to child maltreatment in six studies. Among a national sample of Croatian adolescent moms (*n* = 746), Ajdukovic et al.’ (2018) discovered that welfare receipt was associated with a higher propensity for child maltreatment. In Cheng and Lo’ (2015) study, a lower family income (<$2,000 yearly) was linked to the risk of child abuse (*p* < .01).

Two studies found a strong association between parental *social isolation* and child maltreatment. In Corse et al. (1990), child maltreating mothers (*n* = 26) were less satisfied with available social support (*p* < .01), had less child-rearing help (*p* < .05), and less peer support (*p* < .01) than non-maltreating mothers. Compared to parents who break the cycle of intergenerational child abuse (*n* = 126), cycle maintainers (*n* = 9) had more feelings of loneliness and perceived isolation (*p* < .008).

Parents’ unemployment and housing instability (Slack et al., 2017), reduced satisfaction with housing conditions (Ajdukovic et al., 2018), and maternal companionship support (Price-Wolf, 2014) were some of the other mezzo-level risk factors which were all significantly associated with child maltreatment.

#### Macro-level risk factors

One study identified a macro-level risk factor for child maltreatment, which was the usage of mental health services during pregnancy. This study focused on characteristics of neglectful mothers (Bartlett et al., 2014) and found that adolescent mothers who had previously experienced IPV^1^ and received treatment for mental health issues during their pregnancy had a higher chance of infant neglect (*p* < .001).

**Figure 4.**
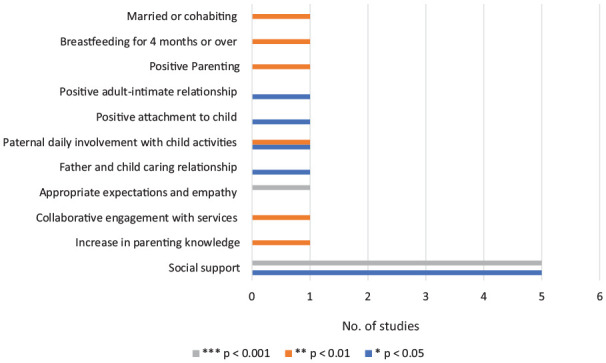
Significant protective factors.

*What protective factors can buffer risk of child maltreatment?* There were 11 significant protective factors found in 18 of the 68 included studies. Of the 18 studies, 15 had high-risk samples and the remaining three studies focused on a low-risk sample. Studies described their samples as high risk but differed in what was considered high or low risk. A pattern was noted among studies that described their sample as high risk and the population had one or more of the following: (i) the presence of two or more individual-level risk factors (e.g., depression, stress, substance abuse), (ii) previous involvement with CPSs, (iii) substantiated current or past record of child maltreatment, and (iv) parental history of childhood maltreatment. A low-risk sample, on the other hand, had no prior child maltreatment history and were not known to CPSs. From these 18 studies, three studies also had comparison groups of high risk versus low risk (Bartlett & Easterborrks, 2015), abuse group versus non-abuse group (Chan, 1994), and high risk versus medium risk versus low risk (Tracy et al., 2018). Figure 4 depicts the protective factors, the number of studies that measured them, and their respective significant levels (*p* values).

In all, 10 studies found social support to be a significant protective factor *(see Discussion, Protective factors for child maltreatment section for varying definitions of social support among studies).* Ajdukovic et al. (2018) investigated the interaction between cumulative effect of risk (low maternal education, low satisfaction with housing conditions, and economic hardship) and social support, finding that when social support was perceived to be high, the effect of cumulative risk on the potential for child abuse was reduced. According to one study (Bartlett & Easterbrooks, 2015), mothers with a history of childhood maltreatment had a decreased risk of infant neglect if they received more social support. Negesh and Maguire-Jack (2016) also found that social support is a protective factor against child neglect and that *perception* of social service availability reduced the likelihood of child physical abuse. Chan’s (1994) study showed that physically abusive mothers had less social support compared to non-abusive mothers.

According to Li et al. (2011), mothers with a high level of social support were 0.29 times less likely to have a child protective report for maltreatment. Price-Wolf (2014) found that higher emotional support was linked to less likelihood of child physical abuse in mothers and fathers, but the link was stronger in women. There was also a link between fathers’ companionship support and a lower risk of child physical abuse, while mothers’ high companionship support was the inverse (risk factor). Companionship support provides a sense of social belonging and includes participation in shared social and recreational activities. For both parents, being married or cohabiting was a protective mechanism against the possibility of child physical abuse.

In two studies, fathers’ involvement in their children’s everyday activities was found to be a protective factor. According to Lee et al.’ (2012) research, daily paternal involvement in child’s activities reduced the chance of child neglect. Lee et al.’s (2012) findings were supported by Slack et al.’s (2011) cross-study comparison. Other protective factors included an increase in parenting knowledge which reduced risk of child maltreatment as well as *actual* maltreatment among teen mothers who had a history of childhood maltreatment (Bert et al., 2009). In the presence of several risk variables, having appropriate expectations of child based on their age and exhibiting empathy toward the child also reduced probability of child maltreatment (DuMont et al., 2012).

### Is There Evidence that Risk and Protective Factors Differ Based on Type of Maltreatment?

In 44 of the 68 research studies, specific type(s) of maltreatment were identified. The remaining 24 studies did not specify a type and instead used umbrella terms of child abuse and neglect or child maltreatment. There were 32 studies on risk factors and 12 studies on both risk and protective factors among the 44 studies. There were 21 studies focused on physical abuse, six studies on neglect, one study on emotional abuse, and one on sexual abuse. The remaining 15 studies focused on various forms of maltreatment: two on physical and emotional abuse; eight on physical abuse and neglect; three on physical abuse, emotional abuse, and neglect; and two on physical abuse, emotional abuse, and neglect.

#### Micro-individual risk factors and maltreatment type

**Figure 5.**
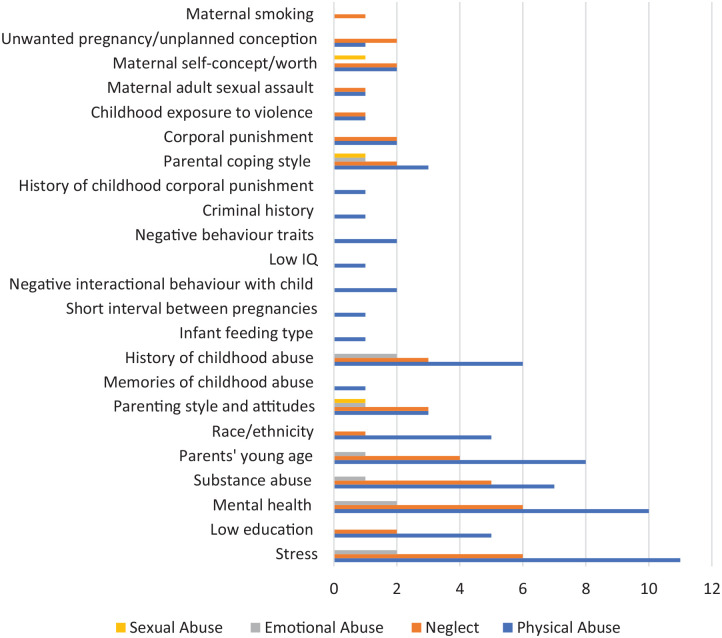
Micro-individual risk factors and type of maltreatment.

As shown in Figure 5, two risk factors were common among all types of maltreatment: (i) parenting style and attitudes to child which included authoritarian style of parenting, lack of enjoyment of child, and not encouraging autonomy in child for physical abuse (Corse et al., 1990); negative view of the child or hostile feelings toward the child for physical, sexual, emotional abuse, and neglect (Milner & Robertson, 1990); and inconsistent discipline and poor supervision and monitoring for neglect and physical abuse (Berkout & Kolko, 2016; Kajese et al., 2011). The second prevalent risk factor was (ii) parents’ coping style and mood quality, which included rigidity, emotion-focused coping, dysregulation in emotion, and this was also common among all types of maltreatment (Lowell & Renk, 2017; Milner & Robertson, 1990).

Several risk factors were found among three types of maltreatment: physical abuse, neglect, and emotional abuse including stress, parents’ mental health, substance abuse, parents’ young age, and parental history of childhood maltreatment. Stress, specifically related to parenting (Berkout & Kolko, 2016; Kim & Maguire-Jack, 2015; Lee et al., 2012; Lowell & Renk, 2017; Macguire-Jack & Negash, 2016; Mash et al., 1983; Price-Wolf, 2014), was found among physical abuse, emotional abuse, and neglect. A common risk factor for two types of maltreatment (physical abuse and neglect) included corporal punishment of child (Berkout & Kolko, 2016; Slack et al., 2011; Whipple & Webster-Stratton, 1991).

Risk factors at the individual level for a single maltreatment type were mostly found for physical abuse and included memories of childhood abuse (De Paul & Domenech, 2000), infant feeding type (bottle feeding and not breast feeding) at the time of discharge from hospital (Kelly et al., 2017), maternal low IQ (Pajer et al., 2014), negative parental traits such as hostility and impulsivity (Price-Wolf, 2014; Rodriguez, 2010), criminal history (Ricci et al., 2003), and parents’ corporal punishment as children (Ross, 1996). Maternal smoking was found to be associated only with child neglect in only one study (Bartlett et al., 2014).

#### Micro-family-level risk factors and maltreatment type

Among family-level micro risk factors, the only commonality between the four maltreatment types was IPV (Banyard et al., 2003; Bartlett et al., 2014; Hunter et al., 2000; McGuigan & Pratt, 2001; Paveza, 1988; Ricci et al., 2003; Ross, 1996; Tracy et al., 2018). Household size which included number of children and number of adults living within a home was common between neglect (Dubowitz et al., 2011) and physical abuse (Chaffin et al., 1996; Connelly & Straus, 1992; Wolfner & Gelles, 1993). Single parent family as a risk factor was found to be common for neglect, child physical abuse, and emotional abuse (Dubowitz et al., 2011; Kelly et al., 2017; Kim & Maguire-Jack, 2015) while marital discord or distress was a common risk factor for sexual abuse (Paveza, 1988), neglect (Zhao et al., 2018) and physical abuse (Whipple & Webster-Stratton, 1991). Engagement of the father in child’s activities and everyday life was a distinct risk factor for physical abuse (Guterman et al., 2009). The only family-level risk factor for sexual abuse that was not detected for other types of maltreatment was the low quality or lack of closeness of the mother–daughter relationship (Paveza, 1988). There were no specific risk factors which were found for emotional abuse.

#### Mezzo-level risk factors and maltreatment type

There were no risk factors for emotional maltreatment found in the studies. Low income and social isolation were two risk factors for sexual abuse, physical abuse, and neglect (Bartlett et al., 2014; Corse et al., 1990). Economic hardship was a common risk factor for physical abuse and neglect (Ajdukovic et al., 2018; Maguire-Jack & Negash, 2016). Within the mezzo level, no distinct indicators of risk for sexual abuse or neglect were found. Physical abuse, on the other hand, had three risk factors independent from other forms of maltreatment. These were parental unemployment (Guterman et al., 2009; Ricci et al., 2003), poorer social status^2^ (Pajer et al., 2014), and maternal companionship support (Price-Wolf, 2014).

#### Macro-level risk factors and maltreatment type

Within the macro-level risk factors, there were no findings related to emotional abuse. Disadvantaged neighborhood was found to be a common risk factor for physical, sexual abuse, and neglect (Drake & Pandey, 1996; Freisthler et al., 2017; Price-Wolf, 2014). A distinct risk factor was found for neglect, which was the use of mental health service. Bartlett et al.’ (2014) study found that one of the risk factors of infant neglect among adolescent mothers is use of mental health services since becoming pregnant.

#### Protective factors and maltreatment type

In all, 13 studies found significant connections between protective variables and types of abuse. Ten of the studies cited social support as a protective factor, while three others found positive parenting and involvement in children’s activities as protective. Social support was protective for physical and emotional abuse and neglect. In the studies that were included, no protective factors for sexual abuse were investigated.

Two studies (Freisthler et al., 2017; Price-Wolf, 2014) found maternal emotional support to be associated a with lower frequency of child physical abuse. Freisthler et al.’ study (2017) showed an association between high emotional support for mothers and a lower potential for child physical abuse and neglect. While Price-Wolf’s (2014) study also found that being married or cohabiting (for mothers and fathers) was also protective against child physical abuse. Paternal companionship support was discovered to be inversely related to child physical abuse. One study (Banyard et al., 2003) showed a link between friendship support as protective of physical abuse and neglect risk. Ajdukovic et al. (2018) investigated mothers’ perception of social support, finding that if mothers felt that they were in receipt of a high level of social support, they were less likely to physically abuse their children. Negash and Maguire-Jack’ (2016) study looked at *perception* of social support and found that it was protective against both physical abuse and neglect.

## Discussion

In this systematic review, most parental risk factors were found at the micro level (both individual and family) including stress (parenting and life pressures), substance misuse, mental health concerns, IPV, childhood maltreatment history, single-parent families, and marital strife. Economic disadvantage and social isolation were the mezzo-level risk factors. At the macro level, the use of mental health services among adolescent mothers since pregnancy increased the risk of infant neglect. Protective factors on a micro-family ecological level were paternal daily involvement with child, positive interactions with child, increase in knowledge about parenting, and breastfeeding for more than 4 months. The most common mezzo-level protective factor was social support. For differences based on maltreatment type, neglect, physical, emotional, and sexual abuse were all linked to IPV, parenting style and attitudes, and parenting coping style. The remaining risk factors were mainly similar across one or more types of maltreatment, while physical abuse, neglect, and sexual abuse each had their own distinct set of risk factors. Social support was found to buffer against physical, emotional abuse, and neglect but not for sexual abuse. A summary of critical findings is presented in Tables 2 and 3.

**Table 2.** Summary of Critical Findings.

Critical Findings of the Review	
Micro-individual-level risk factors	Mental health concerns were the most common risk factor with a significant association with child maltreatment. BPD and PTSD, particularly, were linked to child neglect while maternal depression was linked to child physical abuse Maternal history of childhood maltreatment was the second most common risk factor and recollection and memories of the maltreatment experienced was more significant than its actual occurrence when associated with child maltreatment 18 studies established a link between parental substance use and child maltreatment potential, with the majority of associations with child physical abuse
Micro-level family risk factors	IPV was found to be significantly associated in 12 studies and most linked to neglect and child physical abuse 21 studies measured single-parent family as a risk factor and 9 found a significant association. This co-occurred with multiple other risk factors such as low income and low social support Marital distress was significantly associated in five studies. This includes separation, divorce, and marital disagreement
Mezzo risk factors	Economic deprivation and welfare receipt were linked to physical abuse and neglect and co-occurred with more than 3 minor children at home, maternal smoking, low education, and high stress Social isolation was described differently by the studies with some describing it as lack of child rearing support or lack of peer support and others used maternal perceptions and satisfaction with perceived support
Macro risk factors	Only one study found a significant association between use of mental health services during pregnancy and its association with later child neglect among adolescent mothers who had been victims of IPV Physical abuse, neglect, and emotional abuse shared social support as a protective factor Paternal involvement with children daily was found as protective against neglect No protective factors were found (nor studied in the 68 included studies) specific to child sexual abuse
Protective factors	Fewer protective factors were found and 18 out of 68 studies reported a total of 11 protective factors Social support was the most common and reported in 10 studies. Studies looked at different aspects of social support including availability of support, counseling, emotional support, practical support, and companionship. This was linked with buffering physical child abuse and neglect
Risk factors by maltreatment type—32 out of 68 studies provided a particular type or types of maltreatment and association with child maltreatment	Parenting style and attitudes, IPV, and maternal negative emotional state were common risk factors for all 4 types of maltreatment Physical abuse had the highest number of risk factors and most of these were shared with neglect and emotional abuse including low education, low income, high stress, and maternal childhood history of maltreatment. Other risk factors included mental health concerns, substance use, and more than three minor children One specific risk factor was found for paternal sexual abuse—lack of mother–child closeness
Protective factors by maltreatment type	12 studies specified type of maltreatment and association with protective factors. Social support was a common protective factor for physical abuse, emotional abuse, and neglect. Specific types of social support such as maternal emotional support and friendship support were protective for child physical abuse. Maternal perception of high social support was also found to be protective against child physical abuse. No protective factor for child sexual abuse was found in the included studies

BPD = borderline personality disorder; IPV = intimate partner violence; PTSD = post-traumatic stress disorder.

**Table 3.** Implications of Findings.

Implications of Findings	
Findings related to type of maltreatment	These can help practitioners formulate specific strategies to intervene for based on maltreatment type and the specific risks associated with those
Co-occurrence of risk	Many studies measured more than one risk factor and when delineating type of maltreatment and associated risk, it was clear that there is a high level of co-occurrence of risk especially for child physical abuse, emotional abuse, and neglect. Interventions need to target multiple ecological levels bearing in mind the multitude of risks which may be present at each level
Less research on emotional and sexual abuse	Compared to physical abuse and neglect, studies focusing on parental perpetration of emotional and child sexual abuse were fewer. More studies need to be conducted to get a clearer idea of risk and protection association with these maltreatment types
Protective factors	Far fewer studies reported protective factors compared to risk factors. A more balanced approach in research can help mitigate known risk factors and aid practitioners in developing tools that can buffer multiple risks
Variations in terms and their descriptions across studies	Studies differed greatly in how they described certain maltreatment types (e.g., terms used for physical abuse were interchangeable with harsh discipline or punitive parenting) and in the description of certain risk and protective factors (e.g., social support was described as emotional support in one study and perceived availability of services in another). Uniformity in categorization, description, and definition can help aid research in the field of child maltreatment

### Micro-level Individual Risk Factors

A key finding of parental mental health as a risk factor at the micro-individual ecological level is confirmed by prior research. For instance, parental PTSD symptoms are associated with an increased risk of child maltreatment (Cross et al., 2018) and BPD has been linked to child maltreatment more than any other personality disorder (Battle et al., 2004; Yen et al., 2002). Further to this, a lack of response from caregivers during childhood can result in a decreased ability to regulate emotions as adults (Hughes et al., 2012) which is a key marker of BPD. A prior cohort study (Widom et al., 2009) found that significantly more maltreated children (compared to non-maltreated, demographically controlled children) matched criteria for a BPD diagnosis in adulthood. Consequently, parents with a childhood history of maltreatment may be more likely to develop BPD, increasing the risk of intergenerational transmission of child maltreatment.

Intriguingly, one study’s findings revealed that *memories* of childhood abuse rather than just its occurrence contribute to the likelihood of maltreatment continuance. In a longitudinal study conducted by De Paul and Domenech (2000), adolescent mothers with recollections of childhood physical abuse were more likely to maltreat their newborns than women with a history of childhood physical abuse but no memory of it. While research in this area is sparse to prove that memories of abuse are linked to future abuse potential, one study by Caliso and Milner (1992) found similar results to that of De Paul and Domenech’s (2000) study. Thornberry et al.’ (2013) found a lesser-known link between parents’ experience and timing of abuse in childhood and their future maltreatment potential. Using prospective longitudinal data, researchers discovered that parents with a history of child maltreatment were 2.6 times more likely to maltreat their children when parents were aged between 21 and 30 years (Thornberry et al., 2013). However, there can be underestimation of actual maltreatment as this study only examined validated CPS records to determine maltreatment.

This review also found parental substance abuse to be strongly correlated to child physical abuse and neglect and this can be attributed to a variety of variables, including alcohol’s pharmacological effects on the brain. Parents with identified substance use disorders are consistently reported in the literature to be at a higher risk of child maltreatment, particularly for child physical abuse (Lasslett et al., 2012). Targeting prevention efforts at recovery and avoidance of relapse among substance abusing parents can help buffer the risk of maltreatment.

#### Micro-level family risk factors

IPV was the most common micro family-level risk factor and coincides with findings of prior systematic reviews (e.g., Hindley et al., 2006). This review found that IPV is most closely linked to child physical abuse and neglect (e.g., Bartlett et al., 2014; McGuigan & Pratt, 2001; Ricci et al., 2003) and that it tends to co-occur with other individual-level risk factors such as paternal criminal history (Duffy et al., 2015), maternal depression, and paternal substance abuse (Hunter et al., 2000). Prior research has highlighted the association between poly-victimization (exposure to multiple and traumatic violence; Bidarra et al., 2016) in childhood and subsequent perpetration of IPV and child maltreatment in adulthood (Song et al., 2022). These findings can aid practitioners to provide early prevention services for those children who are exposed to a co-occurrence of risk to prevent future perpetration of both IPV and child maltreatment and provide tailored services to help break intergenerational cycles of violence within families.

One finding of this review is that parents’ single status is associated with an increased risk of child maltreatment. However, single parenthood does not occur in isolation and most of the co-occurring risk factors, as evidenced by this review’s findings and by prior research, can be a by-product of being a single parent, such as low income, low social support, and associated stresses (Berger, 2004; Stith et al., 2009). Single-parent families may benefit from support (e.g., housing, employment, income support) that can help reduce stress resulting from the additional responsibilities of being a single parent.

#### Mezzo risk factors

This review’s finding that social isolation is a key risk factor for child maltreatment is further supported by a body of evidence that associates maternal social isolation with a heightened risk of child abuse (Black et al., 2001; Stith et al., 2009). Consequently, provision of a high level of social support along with satisfaction from that support may help to mitigate child maltreatment risk.

#### Macro risk factors

At the macro level, there was only one significant association with child neglect and use of mental health services during pregnancy among adolescent mothers who had been victims of IPV. This is further linked to an association at the micro level of adolescent mothers and infant neglect and paternal mental health and child maltreatment. Prior research has found that adolescent mothers have a higher chance of developing postpartum depression (Reid & Meadows-Oliver, 2007). A childhood history of maltreatment can also contribute to the development of mental health disorders, psychological distress, and trauma which are established risk factors for child abuse and neglect (Zelenko et al., 2001). Adolescent mothers battling mental health issues may have co-occurrence of multiple adversities which inevitably heighten their potential for child maltreatment.

#### Protective factors for child maltreatment

This review found fewer protective factors than risk factors with social support being the most common protective factor against various risk factors. This finding corroborates previous evidence in this field (e.g., Meng et al., 2018) where social support is consistently considered a buffering mechanism for child maltreatment. This review found that greater emotional support for mothers was correlated with a lower incidence of child physical abuse than that for fathers, despite its association as also protective for fathers (Price-Wolf, 2014). On the other hand, companionship support was found to be protective for fathers whereas companionship support for mothers was associated with an increase in the incidence of child physical abuse. While there is little research on companionship support explicitly, one prior study found that companionship support can act as a conduit for alcohol use, potentially increasing the likelihood of physical abuse (Freisthler et al., 2015). It is also possible that mothers might find socializing to be arduous and stressful when coupled with childcare duties and associated stresses, whereas fathers may find it to be more relaxing.

No protective factors were studied at the macro ecological level within the included studies. A recent literature review with different search criteria than this study (Austin et al., 2020), however, found research evidence which shows macro-level protective factors. Paid family leave policy has been associated with a decrease in infant abusive head trauma (Klevens et al., 2016) and increased minimum wage with a reduction in child protective investigations for neglect (Raisin & Bullinger, 2017). However, these two cited studies were based in California and findings reflect only that population and has limited application to all parents at risk of child maltreatment.

#### Risk factors by maltreatment type

While a considerable overlap of risk factors among types of maltreatment was found, there were a few that were shared by all types of maltreatment, as well as others that were unique to physical, sexual abuse, and neglect.

Parenting style and attitudes (micro-family level) which included a negative attitude and style of parenting was one of the common risk factors for all types. This risk factor was defined differently between studies and included unrealistic expectations of child (DuMont et al., 2012), authoritarian control (Corse et al., 1990), and erratic disciplining (Berkout & Kolko, 2016). A prior systematic review of risk factors also found that poor parenting, such as the ones mentioned earlier, increases the risk of maltreatment recurrence (White et al., 2015). Maternal negative emotional state (Lowell & Renk, 2017) was also found to be common among all maltreatment types. This review, however, only found one study that showed a link between maternal negative emotional state and child maltreatment potential. One issue with this study was that it comprised of a fairly homogeneous sample (Caucasian, married, college degrees) which was considered to be low risk. Nevertheless, maternal emotional state should be investigated further in respect to its links to specific types of maltreatment. The final common risk factor was IPV. A meta-analysis of risk factors found “spousal violence” to have a large effect size but only for child physical abuse and neglect (Stith et al., 2009). Another systematic review (White et al., 2015) supported this finding and found “domestic violence” to be significantly associated with all maltreatment types.

There were also risk factors which were exclusive to one type of maltreatment. Maternal smoking, which was measured in two of the four studies on parental smoking, was only linked to child neglect but mothers in the samples all had co-occurrence of other risk factors. Bartlett et al.’ (2014) study, for example, found that mothers who smoked during pregnancy and neglected their infants also had lower incomes compared to non-maltreating mothers. This review’s findings included a retrospective study (Wu et al., 2004) which examined child protective records and found neglectful mothers of infants also were in receipt of welfare, were single mothers, and had two or more minor children at home in addition to smoking. Previous research has also revealed that child physical abuse is linked to later-life smoking (Yoon et al., 2020), suggesting that these mothers may have experienced maltreatment as children.

The lack of a close relationship between mother and daughter was found as a distinct risk factor for paternal child sexual abuse (Paveza, 1988). Animosity between mothers and daughters can increase the risk of daughters being victims of child sexual abuse by anyone and not just fathers (Schechter et al., 2002). In addition, we found that there was a sparsity of studies on parental risk factors for child sexual abuse within the included studies. This could be indicative of trends in the wider literature on child maltreatment as there are fewer studies on child sexual abuse. More research is needed to see whether there is a link between the quality of mother–daughter relationship and paternal sexual abuse.

This review did not find any exclusive risk factors for emotional abuse but did find some which were also common to other types of maltreatment. A previous systematic review (Black et al., 2002) on risk factors for emotional abuse found only six studies of relevance demonstrating that this type of maltreatment, on its own, is far less studied than physical abuse or child neglect; hence, it becomes difficult to extract risk factors that only occur for emotional abuse and not other types of abuse. A review by Black et al. (2002) identified various parental risk factors for emotional or psychological abuse, including those previously mentioned such as the prevalence of IPV in the home and low income.

#### Protective factors by maltreatment type

Physical and emotional abuse and neglect all had social support as a protective factor, however, we did not find protective factors for sexual abuse. Social support, everyday father’s involvement in children’s lives, and positive parenting behaviors were found to be protective for neglect. A meta-analysis on risk and protective factors for child maltreatment (Austin et al., 2020) had similar findings showing that a higher availability of services (e.g., social services), community involvement, and support from friends and family, can all provide protection against child maltreatment. Further to this, fathers’ sharing of domestic responsibility, providing emotional support to mothers as well as being actively involved with children tend to lower maternal stress and thus the risk of child maltreatment (Dubowitz et al., 2000).

#### Limitations

This systematic review has a few limitations that must be considered. Evidence on risk factors from the mezzo and especially the macro levels is sparse, highlighting the need for more attention and research to be focused on these ecological levels. Another issue is that most studies included in this review focused on mothers, perhaps because they are generally the primary caregivers of children, but this means that findings from these studies may not be applicable to fathers. Furthermore, many included studies were correlational, thus determining causality is not possible. As a result, it is probable that some risk factors are simply indications of risk rather than direct contributors. In addition, much of the included literature focused on child physical abuse and neglect, and this search did not provide many studies on risk and protective variables for child sexual and emotional abuse. Lack of clarity in how abuse is defined, recorded, and measured, as well as difficulty in substantiating parent perpetrated emotional and sexual abuse, may contribute to underreporting and less research on these two types compared to physical abuse and neglect. Furthermore, studies on parental risk and protective factors for child maltreatment included in this systematic review may also not be representative of all studies conducted. While the search was thorough, and every precaution taken to ensure relevant studies were not overlooked, narrowness of the inclusion criteria can contribute to the possible exclusion of other valuable research.

A lack of resources prevented second coding of included studies to establish inter-rater reliability. While double screening is the *recommended* approach, Shemilt et al. (2016) notes that the decision to use a single reviewer in the searching and selecting of studies in a systematic review can “. . .substantively reduce the overall workload and total costs of systematic review production” (p. 11). A methodological systematic review assessing the usefulness of the single-screening approach in study selection concludes that results emanating from this approach are “. . .robust enough to establish this approach as a methodological shortcut” (Waffenschmidt et al., 2019, p. 8). However, to minimize the potential risk of bias emanating from single coding, multiple searches were conducted, and keywords revised to ensure no relevant study was missed.

Lastly, there were few options to synthesize the extensive findings. A meta-analysis was not performed because of heterogeneity among studies. A contentious method of vote counting was thus used. Vote counting has predominantly been used in meta-analytic studies (Bushman & Wang, 2009) and this approach is considered by researchers (Friedman, 2001; Rafaeli-Mor & Steinberg, 2002; Warner, 2001) to be limiting as focus is only on the frequency of significance. This may not be valuable for studies systematically reviewing evaluations of interventions, as using vote counting to differentiate between studies showing benefit (positive studies) or studies showing harm (negative studies) does not reflect accuracy of effect. But this review did not include intervention studies and hence, vote counting was chosen to synthesize findings (Cwikel et al., 2000).

#### Implications and recommendations for research

The findings from this review can provide practitioners tools for formulating strategies based on type of maltreatment and type of risk. Intervention strategies such as intervening during pregnancy, encouraging new and especially young mothers to breastfeed to support positive attachment between mother and child, enhancing parenting knowledge, among others, may reduce the risk of child maltreatment. Addressing parents’ trauma caused by their own childhood history of maltreatment may also reduce risk as it usually co-occurs with multiple risk factors. Encouraging fathers’ involvement in their children’s lives may contribute to reducing maternal stress and increasing perceived and actual social support, thus in turn, lowering the risk of child maltreatment. Further to this, provision of services which reduce parents’ stress, such as help with housing or employment, can help minimize risk, especially for vulnerable parents such as adolescent mothers or single-parent families. For example, social support provision can help protect against co-occurring risk and reduce parents’ stress (micro-individual) by helping them with practical issues such as childcare (micro-family), employment and education (mezzo), housing conditions (mezzo), or protection from a violent partner (micro-family). These findings can further guide interventions targeting vulnerable families with co-occurring risks and provide strategies that can be effective at multiple ecological levels.

The findings of this review on protective factors also mirror some of the intervening strategies of programs targeting reduction of parental risk of child maltreatment. For instance, group-based and community-based parenting programs such as the Durham Family Initiative (Dodge et al., 2004) have been shown to be effective in improving parenting and reducing child maltreatment risk and this may be due to the additional social support element present in such programs.

A recent qualitative study examining effective components in intergenerational child maltreatment interventions for parents found social support, regulating parents’ emotions and, enhancement in parents’ child development knowledge as some of the protective mechanisms that can help break the cycle of maltreatment (Younas & Gutman, 2022). More research, however, needs to be conducted on protective factors to minimize or prevent child maltreatment. Further systematic reviews can examine effective parental interventions and intervening strategies for type-specific maltreatment risk to further knowledge on protective factors in child maltreatment prevention. Specifically, research focusing on delineating risk and protective factors for parent perpetrated emotional and sexual abuse can further knowledge and inform prevention and treatment services. Certain nuances within the findings of this review, for instance, memories of childhood maltreatment history among parents as opposed to occurrence of child maltreatment and its association with further perpetration of child maltreatment, companionship support and its protective effects on fathers versus mothers, among others, require further investigation.

#### Diversity

From the 68 studies, 52 were conducted in United States. Only one study was conducted in each of these countries: India, China, Canada, and New Zealand, while the remaining 12 were from European countries. While child maltreatment remains a global concern, much of the literature and research emanates from the Northwestern part of the hemisphere and this disparity is depicted in the studies included in this systematic review. Further to this, the impact of culture and the way child maltreatment is defined across cultures seems to be lacking within the literature. Given the cultural diversity in populations and the impact of globalization, it seems relevant to contextualize child maltreatment within culture. This may bring to light unique risk and protective factors which can only enrich the study of child maltreatment and its prevention and treatment.

There is also a greater emphasis on mothers and less so on fathers. This is represented in the included studies’ samples with 36 of the 68 included studies which had a population sample of mothers while the remaining 31 focused on both parents and families. No study looked at only fathers. This can pose a variety of problems such as a lack of unique interventions for fathers which are effective and relevant based on the specific needs and strengths of fathers.

## Conclusion

The findings are consistent with previous systematic reviews and reflect well-known parental risk and protective factors within this field. Expanding previous research, this review highlights the overlap between different types of abuse and associated risk and protective factors. IPV was found to be a common risk factor for all types of abuse. The findings suggest that fathers’ lack of involvement with their children, recollections of childhood abuse, and corporal punishment were some of the specific risk factors for child physical abuse. A lack of intimacy between mother and daughter was exclusively linked to an increased likelihood of father-perpetrated sexual abuse. Maternal neglect of infants was exclusively linked to maternal smoking. Neglect, physical abuse, and mental abuse were all linked to the buffering effect of social support, although the way it was classified differed among studies. The results illuminate under-researched areas such as the significance of *memories* of childhood abuse in perpetuating risk (De Paul & Domenech, 2000), the association between parental age and the likelihood of maintaining the cycle of child abuse (Thornberry et al., 2013), and high companionship support as protective for fathers but a risk factor for mothers for physical abuse (Price-Wolf, 2014). While these may be stand-alone findings, they present unique insights and require further research.

Overall, the findings reflect the complex field of child maltreatment and that even after decades of research, evidence is far from conclusive. More research needs to be undertaken to understand these phenomenon to ultimately ensure the effectiveness of prevention services. The findings from this systematic review can serve to help target and tailor services to at-risk parents on multiple ecological levels.

## Supplemental Material

sj-docx-1-tva-10.1177_15248380221134634 – Supplemental material for Parental Risk and Protective Factors in Child Maltreatment: A Systematic Review of the Evidence Click here for additional data file. Supplemental material, sj-docx-1-tva-10.1177_15248380221134634 for Parental Risk and Protective Factors in Child Maltreatment: A Systematic Review of the Evidence by Fatima Younas and Leslie Morrison Gutman in Trauma, Violence, & Abuse

## Notes

1 IPV is defined as an abuse that occurs within a romantic relationship.

2 Using Hollingshead’s (1975) Four Factor Index of Social Status: Education, occupation, sex, and marital status to compute social status.
