# Supplementary material for: Parental Risk and Protective Factors in Child Maltreatment: A Systematic Review of the Evidence
Source: Trauma Violence Abuse. 2022 Nov 30;24(5):3697–714. doi: 10.1177/15248380221134634 (PMC10594837; doi:10.1177/15248380221134634)
Supplement: sj-docx-1-tva-10.1177_15248380221134634 – Supplemental material for Parental Risk and Protective Factors in Child Maltreatment: A Systematic Review of the Evidence [file sj-docx-1-tva-10.1177_15248380221134634.docx]

**APPENDIX A:**

Detailed inclusion criteria for the systematic review.

| Domain | Inclusion Criteria |
| --- | --- |
| Publication | Studies published in peer reviewed journals, book or chapter in book - both reporting findings of empirical study or studies |
| Study Year | 1980-2018 |
| Participants of studies | Parents with children aged 0-17 |
| Focus of study | Quantitative, primary studies that include parental risk and protective factors for child maltreatment |
| Study Methods | Case control and case reviews, longitudinal/cohort, systematic reviews and meta-analysis, cross-study comparisons, cross-sectional |
| Excluded Studies | Opinion pieces, editorials, descriptive/qualitative studies, books which do not report findings of an empirical study, theoretical/conceptual papers, intervention evaluations or intervention studies including meta-analysis or systematic reviews of interventions |

**APPENDIX B:**

Example of search strategy used for one database: PsychInfo

| **#** | **Searches** | **Results** |
| --- | --- | --- |
| 1 | Child abuse.mp. | 31037 |
| 2 | Child maltreatment.mp. | 5380 |
| 3 | Child physical abuse.mp. | 617 |
| 4 | Child sexual abuse.mp. | 6048 |
| 5 | Child neglect.mp. | 4220 |
| 6 | Child emotional abuse.mp. | 43 |
| **7** | **1 or 2 or 3 or 4 or 5 or 6** | **33884** |
| 8 | Risk factor*.mp. | 114306 |
| 9 | Adversity*.mp. | 7613 |
| 10 | Troubled* families*.mp. | 140 |
| 11 | Harsh conditions*.mp. | 58 |
| 12 | Disadvantaged* families*.mp. | 404 |
| 13 | Vulnerable families*.mp. | 249 |
| 14 | Family* difficulties*.mp. | 223 |
| **15** | **8 or 9 or 10 or 11 or 12 or 13 or 14** | **121719** |
| 16 | Protective* factor*.mp. | 13667 |
| 17 | Resilience.mp. | 24010 |
| 18 | Strength*-based.mp. | 2529 |
| 19 | Buffer*.mp. | 11959 |
| 20 | Coping*.mp. | 83238 |
| **21** | **16 or 17 or 18 or 19 or 20** | **125529** |
| 22 | Prevention*.mp. | 127784 |
| 23 | stop*.mp. | 24495 |
| 24 | Discontinue*.mp. | 6880 |
| 25 | Minimi?e*.mp. | 19521 |
| **26** | **22 or 23 or 24 or 25** | **175446** |
| 27 | Parent*.mp. | 275395 |
| 28 | Mother*.mp. | 125956 |
| 29 | Father.mp. | 26771 |
| **30** | **27 or 28 or 29** | **357671** |
| **39** | **7 and 15 and 21 and 26 and 30 (multi-field search – 21 and 30 (Abstract) and 7, 15, 26 (All fields)** | **605** |

**7** = Child abuse OR child maltreatment OR child physical abuse OR child sexual abuse OR child neglect OR child emotional abuse.mp.

**15** = Risk factor* OR Adversity* OR Troubled* families* OR Harsh conditions* OR Disadvantaged* families* OR Vulnerable families* OR Family* difficulties*.mp.

**21** = Protective* factor* OR Resilience OR Strength*-based OR Buffer* OR Coping*.mp.

**26** = Prevention* OR stop* OR discontinue* OR minimi?e*.mp.

**38** = parent* OR mother* OR father*.mp.

**APPENDIX C:**

Data extraction form devised to describe study characteristics from included studies.

**Administrative details**

Data Extraction

- Section A: Administrative Details
  - Name of reviewer
  - Date of Review
  - Document Details
    - Unique Identifier
    - Title of paper
    - Authors of paper
    - Year
    - Name of Journal
    - Is the whole paper used for data extraction or a specific part?
- Section B: Study background
  - What is the purpose of the study?
  - Why was the study done at this point in time?
  - Was the study linked to theoretical/empirical data?
  - When was the study carried out?
  - What are the study hypotheses and/or research questions?
- Section C: Study Focus
  - What area of child maltreatment is the study focused on?
  - What parental risk factors are studied?
  - What parental protective factors (if any) are studied?
  - What is the setting of the study?
  - In which country was the study carried out?
  - Describe in detail the specific phenomenon and factors the study is concerned with.
  - At what time (age of child) were the risk and protective factors studied, if stated?
- Section D: Actual Sample
  - Who is the sample of the study?
  - What was the total number of participants in the study? (Actual sample)
  - What proportion of the sample participated in the study?
  - What parental ages are covered in the sample?
  - What is the ethnicity of the sample?
  - What other useful information is provided about the sample?
- Section E: Study Method
  - Study timing
  - What is the method used in the study?
- Section F: Method-Groups
  - If comparisons made between groups, specify basis of division for making comparisons.
  - How do the groups differ?
  - Number of groups
  - If prospective allocation, what was the unit of allocation?
  - If prospective allocation, what method was used to generate allocation sequence?
  - If prospective allocation, was the sequence concealed?
- Section G: Method-Sampling Strategy
  - Are the authors trying to produce findings representative of a population?
  - What is the sampling frame (if any)?
  - If the study involves studying samples prospectively over time, what proportion of the sample dropped out over the course of the study?
  - If study involves following samples prospectively over time, do authors provide baseline value of key variables?
- Section H: Recruitment and Consent
  - What method was used to recruit people in the study?
  - Were incentives provided to recruit people?
  - Was consent sought?
- Section I: Data Collection
  - Which variables does the study aim to measure/examine?
  - What method was used to collect data?
  - What tools/instruments were used to collect data?
  - Who collected the data?
  - Do authors describe ways in which reliability and validity of data collection methods/tools was addressed?
  - Where was the data collected?
- Section J: Data Analysis
  - What rationale do authors give for the method of analysis for the study?
  - What statistical method was used to analyse the data?
  - Do the authors describe strategies used to control for bias from confounding variables?
  - Do authors describe any ways in which they have addressed the reliability and replicability of data analysis?
  - Do authors describe any way they have addressed the validity/trustworthiness of data analysis?
- Section K: Results and conclusion
  - What are the results of the study?
  - Give details of how the results of the study are represented.
  - Provide details of the authors conclusions

**APPENDIX D:**

**Quality Appraisal Criteria**

- Section A: Population
  - Is the source population well described?
  - Are participants representative of source population?
  - Do selected participants represent eligible population?
  - CASE SERIES ONLY: Was there consecutive and complete inclusion of all participants?
  - Was selection bias minimised in selecting exposure and comparison group?
  - Were confounding factors identified and controlled?
- Section B: Outcomes
  - Were outcome measures and procedures reliable?
  - Were all important outcomes assessed?
  - Was follow-up similar between groups?
  - Was follow up time meaningful?
  - Were strategies to address incomplete follow up described?
- SECTION C: Analysis
  - Was the study powered enough to detect an effect size?
  - Were methods of analysis appropriate and valid?
  - Was the precision of association given or calculable and is the association meaningful?

**APPENDIX E:**

**Included studies’ characteristics**

| **Study** | **Design** | **Child Maltreatment Type and Measure** | **Aims of study** | **Child age** | **Country** | **Study sample and size** | **Complete study/part** |
| --- | --- | --- | --- | --- | --- | --- | --- |
| AjdukoviÄ (2018) | Cross-sectional | Child physical abuse  Child Abuse Potential Inventory (CAPI; Milner, 1986) | Moderating role of social support in the relationship between cumulative risk (socioeconomic status + family economic hardship + higher exposure to stressors) and child abuse potential. As well as relationship between individual risk (e.g., economic hardship) and child abuse potential | 13-16 years | Croatia | 746 mothers recruited from a larger study with mothers and children | Complete |
| Anderson (2018) | Cross-sectional | All child abuse and neglect  Shortened version of CAPI (Milner, 1986) | Exploration of relationships Association of child abuse potential with IPV exposure and psychiatric illness | Not stated | USA | 211 mothers from domestic violence shelters | Complete |
| Appleyard (2011) | Cross-sectional | All child abuse and neglect  CPS records of substantiated and alleged child maltreatment | Relationship between mothers’ childhood history of maltreatment, mental health and substance abuse and association with child abuse and neglect (“offspring victimisation”) | 0-2 years | USA | 499 Pregnant women – both first time mothers and those with children | Complete |
| Banyard (2003) | Cross-sectional | All child abuse and neglect  Conflict Tactics Scale – and Parent Child (CTS and CTSPC; Straus et al., 1995) Check with CPS if parents reported for child abuse | Contributions of unique and common childhood and adult trauma on parenting outcomes in respect to physical child abuse and neglect. Mediating role of maternal depression between trauma exposure and parenting outcomes. Potential protective factors of social support and strong relationships in adulthood. | M 2.69 (SD 1.62) | USA | 152 mothers | Complete |
| Bartlett (2014) | Longitudinal | Child neglect  Conflict Tactics Scale (CTS; Straus et al., 1979) | Using an ecological model of child neglect, influence of characteristics at the level of the child, mother, family, and broader childrearing contexts on adolescent first-time mothers with infants. | Not stated | USA | 383 adolescent mothers with firstborn infant | Complete |
| Bartlett (2015) | Longitudinal | Child neglect  CPS substantiated cases of abuse and neglect | Examination of whether certain factors (positive childhood care, older maternal age, and social support) protect against intergenerational child neglect among high-risk young mothers of infants | 0-1 year | USA | 447 mothers (aged <21 years at birth of first born) | Complete |
| Bartlett (2017) | Longitudinal | All child abuse and neglect  Cumulative records from Department for Child and Families (DCF) | Type-to-type examination of intergenerational child abuse and neglect among adolescent mothers; distinguish transmission to continuity in identifying cases where mother both victim and perpetrator; investigate impact of maternal history of multiple types of maltreatment as child and risk for different types of child maltreatment | 0-8 years | USA | 252 mothers, community sample | Complete |
| Berkout (2016) | Cross-sectional | Child neglect and Child physical abuse (characterised as child-directed aggressive caregiving)  Conflict Tactics Scale (CTS; Straus et al., 1979) | Examination of background and clinical variables among help-seeking parents who were at risk for or had been identified as having engaged in child abuse. Identify characteristics of abusive from non-abusive and explore similarities. Propose model of dysfunction describing relationship between parenting stress, negative affect, positive parenting, and child abuse to assess associations | 9-12 years (M 11.49, SD 3.14) | USA | 195 Parents | Complete |
| Bert, (2009) | Cross-sectional | All child abuse and neglect  CAPI (Milner, 1986) | Examined the intergenerational transmission of abuse among 3 types of mothers (all first time); teen mothers, adult low resource, and adult high resource | 0-1 year | USA | 681 mothers divided into 3 groups, 1) teen mothers <19 years of age and 2) adult>21 years, low-resource mothers, 3) adult, high-resource mothers | Complete |
| Caliso, (1992) | Cross-sectional | Child physical abuse and verbal/emotional abuse  CAPI (Milner, 1986) and CTS (Straus, 1979) | Determine effect of childhood abuse on adult child abuse potential in mothers | Not stated | USA | 90 mothers divided into 3 groups: i) 30 physical child abusers with childhood physical abuse history, ii) 30 non-abusive comparison mothers with childhood physical abuse history, iii) 30 non-abusive mothers with no childhood history of physical abuse. | Complete |
| Chaffin, (1996) | Longitudinal | Child neglect  Child physical abuse  Diagnostic Interview Schedule (DIS; Robins, Helzer, Croughan, & Ratcliff, 1981) | Using data from both Waves I and lI of the National Institute for Mental Health's Epidemiologic Catchment Area survey, 7,103 parents from a probabilistic community sample who did not self-report physical abuse or neglect of their children at Wave I were followed to determine the risk factors associated with the onset of self-reported physical abuse or neglect identified at Wave II. | Not stated | USA | 7,103 parents | Complete |
| Chan, (1994) | Cross-sectional | Child physical abuse  CPS records - substantiated | Examines the role of parenting stress and maternal social support in physical child abuse in Hong Kong. | Not stated | Hong Kong | 74 mothers; 37 abusive and 37 non-abusive comparison mothers | Complete |
| Chang (2008) | Cross-sectional | All child abuse and neglect  CPS records - substantiated | Examine types of maltreatment and child and parent sociodemographic and behavioural characteristics among Cambodian refugee families. | 0-18 years | USA | 71 parents with 243 children (average of 3.4 children per family) | Complete |
| Cheng (2015) | Longitudinal | All child abuse and neglect  CPS records – substantiated | Explore impacts of parental receipt of social services and caseworkers' and parents' collaborative engagement on substantiated child maltreatment re-report | 0-17 years | USA | 5676 parents with prior CPS reports of substantiated child abuse (parents with substantiated re-report 2368) | Complete |
| Christensen (1994) | Longitudinal | Child neglect  Child physical abuse  Tennessee department of Human Service records – alleged reports | Association of parental low self-esteem with child maltreatment | 0-4 years | USA | 471 pregnant women | Complete |
| Connell (2009) | Longitudinal | Child neglect  Child physical abuse  Child sexual abuse  CPS substantiated records | Compare rates of maltreatment among children following parental reunification between children in foster care due to maltreatment and those in foster care for other reasons. Assess effects of child, family, and case characteristics on rate of re-maltreatment. | 0-16 years | USA | 3226 Parents and children | Complete |
| Connelly (1992) | Cross-sectional | Child physical abuse  CTS (Straus, 1979) | Examine association of maternal age and risk of child physical abuse using a nationally representative sample | Mean age 8.8 years | USA | 1997 mothers; 251 abuse group, 1746 comparison group | Complete |
| Corse, (1990) | Cross-sectional | Child physical abuse  CPS – substantiated | Compare the social networks of mothers in families identified as abusive and mothers in control families’ relationships between social networks, parenting beliefs and practices and child abuse. | Abuse group mean 7.25, comparison group M 7.13 | USA | 52 mothers | Complete |
| de (2000) | Longitudinal | Child physical abuse  CAPI (Milner, 1986) | Determine whether adolescent mothers of new-borns are at higher risk for child abuse than adult mothers of new-borns and to examine whether adolescent mothers with memories of child maltreatment have a higher risk for child abuse. | Initial assessment when mothers' 5-6 months pregnant, follow up when child 1, 6, 12 and 18 months of age | Spain | 48 mothers (24 adolescents and 24 adult mothers) divided into 3 groups; 23 mothers who had severe physical abuse as child (SPP group), 12 mothers who had severe physical childhood abuse with physical damage (PD group) and 13 mothers who had childhood emotional abuse (EW group) | Complete |
| Dixon (2009) | Longitudinal | All child abuse and neglect  Child Protection professionals for suspected or actual physical, sexual, or emotional child abuse and neglect | Investigate factors (parenting styles, individual risk factors) associated with continuation and discontinuation of intergenerational transmission of child abuse within 1st year of child's life. | 0-1 years | England | 4351 families | Complete |
| Doidge (2017) | Cohort | All child abuse and neglect  Self-report questionnaire | Explore child, parent, and family risk factors for child maltreatment to identify high-risk groups and independent predictors of each type of child maltreatment. | 0-27 years | Australia | 2443 infants | Part |
| Doris (2006) | Longitudinal | All child abuse and neglect  New York State Central Register of Child Abuse and Neglect (SCR) - substantiated reports of maltreatment | Child welfare data were examined to explore relationship between mothers' cocaine use (prenatal) and subsequent child welfare outcomes | 0-3 years | USA | 152 mothers and 152 infants | Complete |
| Drake, (1996) | Cross-sectional | Child neglect, child physical and sexual abuse  Missouri’s Child Abuse and Neglect database for substantiated and alleged reports of child maltreatment | Explore relationship between neighbourhood poverty and three different types of child maltreatment: neglect, physical abuse, and sexual abuse | Under 18 years | USA | 481722 families within select zip codes based on income (low or moderate) | Complete |
| Dubowitz (2011) | Longitudinal | Child neglect and physical abuse  CPS records – reports only | Explore association of multiple levels of risk factors (child, parent, family, community) to examine antecedents and outcomes of maltreatment. | Start of study average age of infant 14 months; followed till child 12 years | USA | 224 Parents (mother or father) and 224 children | Part (child outcomes/child variables excluded) |
| Duffy (2015) | Case-control | All child abuse and neglect  CPS reports | Explore relationship between parental risk factors and substantiating status and number of CPS reports in families | Median age at first CPS report 5 months (range 0-42 months) | USA | 131 high-risk families receiving services for child abuse prevention | Complete |
| DuMont (2012 – book chapter) | Secondary data analysis | All child abuse and neglect  NYS Statewide Automated Child Welfare Information System – substantiated records | Explore the influence of promotive factors in achieving resilience to child abuse and neglect among at-risk mothers | Not stated | USA | 524 Mothers taken from control group data from a longitudinal RCT of Healthy Families New York | Part - only maternal characteristics - not child characteristics |
| Freisthler (2017) | Cross-sectional | Child neglect and Child physical abuse  Physical abuse – CTSPC (Status et al., 1995) Neglect – Multidimensional Neglectful Behaviour Scale (Straus and Kinard, 2004) | Assesses the relationship between indicators of drug demand and drug supply on physical abuse, physical neglect, and supervisory neglect in a general population sample. | M 6.71 years SD 3.62 | USA | 2597 parents | Complete |
| Fuller (2003) | Longitudinal | All child abuse and neglect  Illinois Child Abuse and Neglect Tracking Systems Database – all cases opened for investigation | Examination of factors that are predictive of short-term maltreatment recurrence among CPS cases among cases of parents with alcohol and drug use | Not stated | USA | 95 Parents with prior CPS reports of child maltreatment | Complete |
| Grumi (2017) | Cross-study comparison | All child abuse and neglect  Families referred to CPS for maltreatment | Exploration of relationships risk and protective factors assessment by CPS to place children in foster care | n=328; M 8.41 years (SD 4.89) range 0-17 years | Italy | 328 families with 313 fathers and 323 mothers (Italian versus immigrant families) | Part - Not considered child variables |
| Guterman (2009) | Cross-sectional | Child physical abuse  self-report and observational measures assessed aspects of parenting behaviour that might foreshadow or indicate risk for physical child abuse | Retrospective study aimed to examine the presence/absence of a set of risk and protective factors among Italian and immigrant families for whom Child Protection Services intervened with the child's placement in out of home care | Not stated | USA | 1480 parents with maternal CPS maltreatment record | Complete |
| Haapasalo, (1999) | Cross-sectional | All child abuse and neglect  CPS substantiated records | Abusive and/or neglecting mothers whose child had been under the supervision of the child protection services compared with mothers who had never had any contact with such an agency. The specific aims were to examine the differences between the two groups of mothers in their reports of childhood maltreatment experiences and to test whether the mothers' self-reported childhood experiences could explain maltreatment directed at their own children | CPS group (n=25) M 12.68 years; comparison group (n=25) M 11.88 years | Finland | 50 mothers and 50 children divided equally into CPS report groups and non-CPS report group | Complete |
| Herrenkohl (2013) | Longitudinal | Child physical abuse  described as 'abusive disciplining'  Parents’ self-report | Association of parents' history of physical punishment in childhood and physical abuse of offspring | Children pre-school age at start of study and last follow-up when children aged 30 years | USA | 268 children followed over 30-year period | Complete |
| Hunter (2000) | Cross-sectional | Child physical and psychological abuse  CTSPC (Starus et al., 1995) | Description of risk characteristics of abusive parents from an Indian village. | 0-16 years | India | 395 mothers | Complete |
| Kajese (2011) | Secondary data analysis | Child neglect (leading to or contributing to death) and Child physical abuse (leading to/contributing to death)  Kansas CPS and county records | Describe epidemiology of child abuse homicides to identify risk factors among abusive parents. | 0-16 years | USA | parents of 170 children who had died (from CPS records of maltreatment) | Complete |
| Kelly (2017) | Case-control | Child physical abuse (abusive head trauma)  Hospital records | Examine data routinely available to perinatal healthcare providers, to identify factors associated with the occurrence of abusive head trauma, and to contribute to evidence that could inform targeted prevention programs. | 0-2 years | New Zealand | Mothers (142 cases and 550 controls) | Complete |
| Kim (2015) | Secondary data analysis | Child neglect, Child physical abuse, Child emotional/psychological abuse  CTSPC (Straus et al., 1995) | Association of certain risk factors (parenting attitudes, relationships, demographic data, mental and physical health, etc.) and child maltreatment behaviour. | 9 years | USA | 2991 mothers from a longitudinal study (only wave 5) | Complete |
| Lee (2012) | Cohort | Child neglect  CTSPC (Straus et al., 1995) | To examine the association of paternal depression with risk for parental neglect of young children. | Risk factor assessment when child 3 years old, child neglect assessment when child 5 years (neglect in the past year) | USA | 1089 families | Complete |
| Lesnik-Oberstein, (1995) | Cross-sectional | Child emotional/psychological abuse  CTS (Straus et al., 1979) | Identify risk factors for psychological abuse of children | Abused group Mean age 1.5 years (SD 2.6); comparison group M 3.1 years (SD 3.9) | Netherlands | n= 344 participants (mothers and children)  comparison group consisted of 128 children and their mothers. The psychologically abused group consisted of 44 children and their mothers. | Complete |
| Li (2011) | Longitudinal | All child abuse and neglect  CPS reports | Explore both risk and protective factors of child maltreatment among at risk elementary school children. | From age 4-5 years to age 8-9 years | USA | 405 Mothers and children | Complete |
| Lowell (2017) | Cross-sectional | Child neglect  Child physical abuse  Child emotional/psychological abuse  CAPI (Milner 1986, 1994) | Investigation impact of child risk factors (behavioural and emotional difficulties) for child maltreatment potential among mothers with young children. | 1.5- 5 years | USA | 158 mother and child dyads | Complete |
| Maguire-Jack (2016) | Cross-sectional | Child neglect  Child physical abuse  CTSPC (Straus et al., 1995) | Examines the relationship between aspects of social service availability and child maltreatment. Specifically, estimate whether service availability, accessibility, and receipt are associated with physical child abuse and neglect. | Not stated | USA | 1053 parents | Complete |
| Mash, (1983) | Cross-sectional | Child physical abuse  Abusive mothers referred from a child abuse program | Compare interactions of abusive and non-abusive mothers with their children to identify behavioural and interactional risk factors | Abused sample mean age 55.4 months; non-abused sample mean age 59.3 months | Canada | 72 participants - 2 groups of 18 mother-child dyads (abused and non-abused group) | Complete |
| McGuigan (2001) | Cohort | Child neglect  Child physical abuse  Child emotional/psychological abuse  Oregon child protective services agency – 6% confirmed cases, others reported | Relationship between domestic violence and each type of child maltreatment (neglect, physical and emotional abuse) occurrence from birth till child 5 years old | Birth till child 5 years old | USA | 2544 families | Complete |
| Metzner (2017) | Cross-sectional | All child abuse and neglect  Self-reporting questionnaire | Examined the characteristics of fathers in psychosocially stressed families and associations between paternal risk factors (PRFs: mental health disorder, physical health disorder, young paternity, unemployment, absence of father) and family risk factors (FRFs: problematic financial situation, problematic housing situation, social isolation) for child maltreatment. | Not stated | Germany | 506 at-risk families | Complete |
| Milner, (1990) | Cross-sectional | Child neglect  Child physical abuse  Child sexual abuse  CAPI (Milner, 1986) | Investigate psychological and social characteristics of parents who have abused their children physically or neglect their children | Not stated | USA | 150 parents - 75 child abusers, 75 non-abuser comparison group | Complete |
| Negash (2016) | Cross-sectional | Child neglect  Child physical abuse  CTSPC (Straus et al., 1995) | Availability of social services (within the context of social support) and its' association with reduction in child abuse and neglect | Not stated | USA | 1050 parents | Complete |
| Pajer (2014) | Cross-sectional | Child physical abuse  CAPI (Milner, 1986) | Determine whether psychopathology, exposure to maltreatment, preparedness for childbearing, substance use disorders (SUDs), IQ, race, and socioeconomic status were associated with the potential for child abuse in nonpregnant adolescent girls. | Not stated | Not stated | 195 Adolescent girls of childbearing age | Complete |
| Paveza, (1988) | Case-control | Child sexual abuse (father to daughter)  Families obtained from: i) CPS referrals ii) court referrals iii) self-referred | Characteristics of fathers who sexually abuse their daughters to identify risk factors to predict such abuse. | 5–18-year-old girls | USA | 34 mother-daughter dyads in abused group and 68 dyads in control group | Complete |
| Price-Wolf (2014) | Cross-sectional | Child physical abuse  CTSPC (Straus et al., 1995) | Examined relationship between social support, collective efficacy, and child physical abuse and compared the impact on mothers versus fathers | Mean 6.7 years (SD 3.6) | USA | 3023 parents | Complete |
| Ricci (2003) | Secondary data analysis | Child physical abuse (Abusive head trauma)  Maine Hospital records | Characteristics of parents of children who have died due to abusive head trauma | 2 weeks to 17 months | USA | Parents of 19 children with abusive head trauma | Part - child risk factors not considered |
| Rodriguez (2010) | Cross-sectional | Child physical abuse  CAPI (Milner, 1986) | Investigated whether parenting-relevant cognitions (e.g., hostility, stress and coping skills) would predict child abuse potential | < 12 years, mean age 5.86 years | USA | 363 parents; 53 fathers and 310mothers | Complete |
| Rodriguez (2015) | Cross-sectional | Child physical abuse  CAPI (Milner, 1986) | Explored role of cognitive processes (negative child attributions and dispositional empathic ability) in predicting maternal child physical abuse risk | 6–9-year-old children (mean age 7.46) | USA | 95 mother-child dyads | Complete |
| Romero-Martinez, (2013) | Cross-sectional | Child physical abuse  CAPI (Milner, 1986) – Portuguese version (Gomes, 2010) | Explored role of parent's gender, timing of childhood abuse and socio-demographic variables on the relationship between parents' history of childhood physical abuse and current risk for children. | Not stated | Portugal | 920 parents (414 fathers, 506 mothers) | Complete |
| Ross, (1996) | Cross-sectional | Child physical abuse  CTS (Straus et al., 1979) | Estimated the gender-specific probability of a violent spouse also physically abusing his or her child within a representative sample. | 0-18years | USA | 3363 parents (or single parent) of child under 18 living at home | Complete |
| Schick (2015) | Cross-sectional | All child abuse and neglect  Self-report | Examined prevalence and risk factors of various types of CM in a population-based representative sample of native and immigrant adolescents in Switzerland | Adolescent 13-20 years, mean age 15.04 | Switzerland | 6787 adolescents | Complete |
| Sedlak, (1997) | Secondary data analysis | All child abuse and neglect  From the National Incidence Study of Child Abuse and Neglect (NIS-2) – representative database of child abuse and neglect cases – all reports | A large database of child abuse and neglect was analysed to identify demographic risk factor for child abuse and neglect | 0-17 years | USA | 6033 children:  nationally representative sample of 2,235 children who met the Harm Standard were combined with a comparison database of 3,798 nationally representative non-maltreated children obtained in the U.S. Bureau of Census Current Population | Complete |
| Cantos (1997) | Cross-sectional | Child physical abuse  Mothers referred to child abuse prevention program at Columbia-  Presbyterian Medical Center in New York City – substantiated | Mothers who had physically abused their children were assessed to determine whether these mothers had a general coping skills deficit. Abusing mothers were compared to non-abusing mothers of conduct problem children. | Not stated | USA | Total mothers = 33; Abusing mothers n=17 versus non-abusing mothers n =16 | Complete |
| Slack (2011) | Cross-study comparison | Child neglect  CPS maltreatment report | Cross-study comparison to identify risk and protective factors for child neglect | Not stated | USA | 2622 parents (Across 3 longitudinal studies) | Complete |
| Slack (2017) | Cross-sectional | All child abuse and neglect  CPS maltreatment report | Exploratory study examines combinations of income-tested welfare benefits and earnings, as they relate to the likelihood of child maltreatment investigations among low-income families with young children participating in a nutritional assistance program. | 0-2 years | USA | 1065 parents | Complete |
| Thornberry (2013) | Longitudinal | All child abuse and neglect  CPS records at the New York State Office of Children and Family Services - substantiated | Investigate whether safe, stable, and nurturing relationships can interrupt cycle of child abuse | 14-30 years | USA | 711 adolescents | Complete |
| Thornberry (2014) | Longitudinal | All child abuse and neglect  Substantiated cases from CPS records | Investigate adolescent risk factors, measured at both early and late adolescence, for involvement in child maltreatment during adulthood. | 13 years - 31 years | USA | n=816 - G1 Parents, G2 Adolescents | Complete |
| Tracy (2018) | Longitudinal | Child physical abuse  Child emotional/psychological abuse  Maternal self-reports | Examined whether maternal social support in early childhood, and [also paternal involvement in middle childhood] could prevent the intergenerational transmission of abuse | prenatal till child 8 years old | England | From 14,541 pregnant women, 13,988 live births | Part - not concerned with offspring violence - 2 separate analysis in study |
| Valentino (2012) | Secondary data analysis | All child abuse and neglect  Self-reports/questionnaires | Community violence and authoritarian parenting attitudes were evaluated as predictors of the intergenerational continuity of abuse, and the moderating effect of African American race was examined. | 0-18 years | USA | 70 first-time adolescent mother and child dyads | Complete |
| Whipple, (1991) | Cross-sectional | Child physical abuse  CPS involvement | Role of several psychosocial stressors, individual components of stress and support in physically abusive and non-abusive families with conduct-problem children. |  | USA | 123 families (divided into two groups of abusive/non-abusive parents) | Complete |
| Wolfner (1993) | Cross-sectional | Child physical abuse  CTS (Straus et al., 1979) | Survey of national sample of parents to identify characteristics of physically abusive parents | 0-18 years | USA | National sample of 5941 parents | Complete |
| Wu (2004) | Cohort | All child abuse and neglect and threatened harm - a wilful act that is intrinsically harmful or dangerous which could clearly and immediately result in injury or harm.  Substantiated records from Florida CPS | Identified perinatal and sociodemographic risk factors associated with maltreatment of infants up to I year of age | Prenatal to 1 year old infant | USA | 189055 Mother and infant dyads | Complete |
| Zhao (2018) | Longitudinal | Child neglect  CTSPC (Straus et al., 1995) | Identified the change of prevalence and influencing factors for child neglect in a rural area of Anhui province through the 2-year follow-up study. | 7-16 years | China | 816 children | Part - not considered child variables such as child's coping style and social anxiety |
| Zuravin, (1987) | Cross-sectional | Child neglect and physical abuse  Baltimore social services – substantiated cases | Explored relationship between contraception use, unplanned pregnancies and child abuse and neglect | 0-12 years | USA | 518 single mothers receiving public assistance | Complete |
